# Safety Evaluation of Ultrasonic‐Assisted OSA‐Modified Tamarind Seed Gum for Food Applications

**DOI:** 10.1002/fsn3.71079

**Published:** 2025-10-17

**Authors:** Adan Naeem, Rizwana Batool, Mahwash Aziz, Aysha Sameen, Aiza Zafar, Faiza Azmat, Bakhtawar Saleem, Qamar Sajjad, Awais Raza, Agoura Diantom

**Affiliations:** ^1^ Department of Food Science and Technology Government College Women University Faisalabad Faisalabad Pakistan; ^2^ Department of Nutritional Sciences and Environmental Design Allama Iqbal Open University Islamabad Pakistan; ^3^ National Institute of Food Science and Technology University of Agriculture Faisalabad Faisalabad Pakistan; ^4^ University Institute of Diet and Nutritional Sciences The University of Lahore Lahore Pakistan; ^5^ Department of Food Science and Technology Universite de Lomé Lomé Togo

**Keywords:** biochemical parameters, hematological parameters, safety, TSG, ultrasonic‐assisted OSA–TSG

## Abstract

Evaluating the safety profile of substances used in food products is essential. Ultrasonic‐assisted octenyl succinic acid‐modified tamarind seed gum (OSA–TSG) has shown potential as an emulsifier and stabilizer in a variety of food applications; however, its safety profile has not yet been thoroughly investigated. In the present study, the acute oral toxicity of both pure tamarind seed gum (TSG) and ultrasonic‐assisted OSA–TSG was evaluated in Sprague Dawley rats over 21 days. The purpose of the study was to assess the safety of these gums with respect to potential adverse effects and mortality following a single oral dose of 300 and 2000 mg/kg body weight. Body weight and feed intake were monitored at 0 and 21 days, respectively, and significant effects were observed in the TSG and ultrasonic‐assisted OSA–TSG‐treated groups compared to the control group. No adverse effects were seen in hematological and biochemical parameters in either treatment group. Furthermore, no macroscopic or histological alterations were found in vital organs (heart, brain, liver, and kidney) upon necropsy that could be attributed to the administration of test substances. In conclusion, both pure and ultrasonic‐assisted OSA–TSG exhibited an LD_50_ value greater than 2000 mg/kg, indicating a high margin of safety. Therefore, these gums may be considered safe and viable ingredients for use in the development of a diverse range of food products.

## Graphical abstract

Ultrasonic‐assisted OSA modification enhances tamarind seed gum functionality and safety for food applications. Subacute toxicity study confirms biocompatibility, supporting its potential as a novel food ingredient.

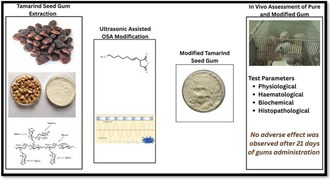

## 1 Introduction

Toxicity refers to the degree of adverse effects due to interactions between a toxicant and biological cells. The chemical composition of toxicants can influence various biological structures, including cell surface, extracellular matrix, and subcellular regions of tissues. Therefore, evaluating the toxicity of the compound is crucial before its application as a food or pharmaceutical excipient (Malik et al. 2022). Substances commonly found in food are generally considered safe based on a long history of consumption, which establishes a precedent for their safety. However, novel functional ingredients without such a history require rigorous safety assessments before their introduction to the market. The safety evaluation of new functional ingredients should adhere to the same principles used for other substances and must reflect their intended usage levels. A comprehensive safety evaluation should consider both the functional ingredient itself and the final food product in which it is incorporated, taking into account the potential risks and benefits associated with each component. For substances lacking a prior history of safe consumption, an in‐depth and systematic review of scientific literature regarding their anticipated biological effects is essential (Fabiansson 2014).

Tamarind seed gum's xyloglucan is composed of long chains of β(1–4)‐linked glucose molecules; approximately 75% are substituted with α(1–6)‐linked xylose units. Some of these xylose side chains are further attached to galactose molecules via β(1–2) bonds. Four distinct types of oligosaccharide units repeat as the primary structural components of the polysaccharide found in tamarind seeds. According to dietary intake assessment, consumption of the tamarind seed gum (TSG) as a stabilizer and thickener in various foods (such as mayonnaise, flour products, soup, sauces and condiments, ice cream, desserts, dressings and fruit preserves, pickles, spreads and fillings, and beverages) may reach 91 mg/kg body weight/day among extensive users of these foods, according to a dietary assessment (Heimbach et al. 2013). The United States Food and Drug Administration (FDA) granted the tamarind seed polysaccharide products generally recognized as safe (GRAS) classification in 2014, allowing for its marketing in the US (Yamatoya et al. 2020). Additionally, the Joint Expert FAO/WHO Committee on Food Additives (JECFA) established an acceptable daily intake (ADI) of “not specified” for tamarind seed galactoxyloglucan, indicating a high level of safety based on evidence from rodent toxicity studies and the absence of genotoxic, reproductive, or developmental toxicity concerns (WHO 2019).

Native tamarind seed gum is widely used in food and pharmaceutical applications due to its excellent water solubility, mucoadhesiveness, gelling ability, and thickening properties. However, for certain advanced applications, its inherent properties may be insufficient, particularly where substantial emulsification or interfacial activity is required (Malviya et al. 2021). By chemically grafting hydrophobic octenyl succinic anhydride (OSA) moieties onto the polysaccharides' backbone, the modified gum acquires amphiphilic characteristics, presenting both hydrophilic and hydrophobic segments. The only alkenyl succinate that has been approved for use in food applications at concentrations below 3.0% is OSA, which plays a crucial role in stabilizing polysaccharides in the gastrointestinal tract. The incorporation of OSA into spherulite granules increases the hydrophobicity of the polysaccharide molecules. However, the hydrophilic character of the molecule retains due to the presence of an unsubstituted hydroxyl moiety, resulting in amphiphilic OSA‐esterified polysaccharide (Sweedman et al. 2013). According to Kou et al. (2023), this significantly improves its ability to act at oil–water interfaces, enhancing its performance as an emulsifier and stabilizer in food emulsion.

Various technological approaches have been investigated for polysaccharides, among which ultrasonication has emerged as a promising and environmentally friendly technique, primarily employing acoustic energy to generate highly confined areas for energy dissipation. This increases the molecular interactions in a solution, which eventually accelerates the processes as a whole. OSA‐esterified polysaccharides exhibit a broad range of molecular structures, giving rise to distinct functional properties. The product quality, solubility, and viscosity can be changed by adjusting the reaction conditions with OSA and the structural features of OSA‐esterified polysaccharide, such as degree of substitution, degree of branching, chain‐length distribution, and the molecular size distribution (Gautam et al. 2023). Furthermore, ultrasonic‐assisted OSA modification markedly enhanced key functional parameters including swelling index, water‐ and oil‐holding capacity, emulsifying capacity, and overall emulsion stability (Naeem et al. 2025). For successful commercial scaling, modified tamarind seed gum still requires safety and regulatory evaluation. However, the current research was carried out to investigate the safety of both pure and ultrasonic‐assisted OSA‐modified TSG using a rat model.

## 2 Materials and Methods

### 2.1 Test Material

Tamarind seed was procured from the local market of Faisalabad, Pakistan, and the study was conducted at Pakistan's Government College Women University Faisalabad. Standards and reagents were acquired from Sigma‐Aldrich (Japan) and Merck (Germany). Male Sprague Dawley rats from the National Institute of Health (Islamabad) were obtained for the efficacy experiments. The diagnostic kits that were utilized came from Cayman Chemicals (Estonia), Bioassay (Germany), and Sigma‐Aldrich.

### 2.2 TSG Extraction

After being cleaned under running water and dried at 40°C for 24 h, the tamarind seeds were stored in clear bottles covered with aluminum foil to protect them from light and heat. The seeds were then roasted, decorticated using a seed decorticator, and ground into powder following the method of Reis et al. (2016). The defatted tamarind seed powder was mixed with distilled water at a 1:10 (w/v) ratio and stirred vigorously for 10 min at 800 rpm. The pH of the mixture was adjusted to 9.5 using 1 N sodium hydroxide. The solution was then heated to 20°C–22°C and stirred for 30 min at level seven. Centrifugation was performed to separate the alkaline extract and supernatant containing solubilized protein for 15 min at 4°C and 4370 rpm. To adjust the pH to 6.8, the resulting mass was transferred to a 2 L beaker containing distilled water at a 1:30 (w/v) ratio and 1 N hydrochloric acid. The mixture was left to stand at 20°C for 24 h to facilitate gum release. A second centrifugation was conducted for 15 min at 10°C and 5240 rpm to extract the remaining material. The 96% ethanol was added in a 1:2 (v/v) ratio to extract the gum from the supernatant by stirring continuously for 10 min. After that, the mixture was let to stand for two more hours. Finally, the extracted TSG was ground into a powder using a grinder after being dried at 45°C in a hot air oven until it reached a constant weight (Crispín‐Isidro et al. 2019).

### 2.3 Yield

TSG's yield was determined following the procedure of Crispín‐Isidro et al. (2019)

(1) $$\text{Yied}\;\left(\%\right)=\frac{\mathrm{TSG}\;\text{weight}\;\left(\mathrm{dry}\;\text{basis}\right)}{\;\text{Tamarind seed weight}\;\left(\mathrm{dry}\;\text{basis}\right)}\times 100$$

### 2.4 Purity Analysis of TSG

Standard qualitative tests for carbohydrates, proteins, lipids, tannins, and saponins were conducted on extracted tamarind seed gum to assess its purity (Malviya et al. 2021).

#### 2.4.1 Carbohydrate

The solution of tamarind seed gum (TSG) was combined with Benedict reagent and then heated in a water bath. The reddish‐brown precipitation in the solution indicated the presence of carbohydrate.

#### 2.4.2 Protein

The sample (0.5 g) was mixed with distilled water. The mixture was then combined with an equal amount of Biuret reagent. Afterward, it was mixed thoroughly and let it set for 5 min. The appearance of a purple color indicated the presence of protein.

#### 2.4.3 Lipids

Sudan Red III was added to a 1% gum solution. The variation in the color of the solution indicated the presence of lipids.

#### 2.4.4 Tannin

The TSG solution was combined with eight drops of FeCl_3_. The emergence of greenish‐black or bluish residue indicated that tannins were present.

#### 2.4.5 Saponins

The TSG solution was combined with water and shaken vigorously until foam formed that indicated the presence of saponins.

### 2.5 Preparation of Ultrasonic‐Assisted OSA–TSG

A 35% (w/v) gum suspension was prepared by dissolving 2 g TSG in distilled water, and the pH of the suspension was adjusted to 8.0 using NaOH solution. During the alkalization process, the temperature of the suspension was maintained at 35°C for 20 min. After adding 3.0% of OSA solution (on a dry gum basis), the pH was maintained at 8.0. The mixture was then diluted three times with anhydrous ethanol. Using a probe‐type ultrasonic processor under consistent settings, the suspensions underwent ultrasonic treatment for 60 min. Each cycle consisted of 5 min of sonication, followed by a 25 min rest period, during which the temperature was allowed to return to 35°C under continuous stirring. The 6 h were required for the combined ultrasonication and esterification process. After the reaction, the suspension was neutralized to pH 7.0 using 6.5 mL of 1 M HCl solution, followed by vacuum filtration through a filter paper. The residue was washed four times, once with ethanol solution (95% v/v) and a further three times with distilled water to remove residual reactants. The final product was dried and sieved through a standard 100‐mesh sieve (Zhang et al. 2020).

### 2.6 Degree of Substitution

The octenyl succinate substituent was utilized to replace hydroxyl groups, creating the TSG–OSA conjugate. The standard degree of substitution (DS) was used to represent the amount of substituents along the TSG chain. Titrimetric analysis was used to determine the octenyl succinic concentration in ultrasonic‐assisted OSA–TSG conjugate. The unreacted OSA was removed by rinsing the esterified gum (1 g) with 20 mL of methanol. The obtained solution was agitated for 30 min while suspended in 4 mL of 0.1 M HCl. The AgNO_3_ was added in solution; as a result, chloride ions were formed. After this, a glass filter was used to filter the solution in order to separate the chloride ions and rinsed with distilled water. Distilled water was used to re‐disperse the sample. Later, it was boiled in a water bath for 20 min and titrated with 0.1 M NaOH solution with phenolphthalein. As a control, unmodified TSG was also titrated. The percentage of OSA in OSA–TSG and ultrasonic‐assisted OSA–TSG and the degree of substitution of OSA were calculated according to (Equations 2 and 3) (Pourramezan et al. 2022).

(2) $$\mathrm{OSA}\;\left(\%\right)=\left(v1-v2\right)\;0.1\times 21/W$$

(3) $$\mathrm{DS}=1735\times \%\mathrm{OSA}/\left(210-209\right)\times \%\mathrm{OSA}$$

where

v_1_ = Volume in mL of NaOH used for TSG_OSA_.

v_2_ = Volume in mL NaOH used for TSG.

W = Weight of TSG_OSA_ in grams.

% OSA = OSA weight percentage in TSG_OSA_.

### 2.7 Biosafety Assessment

The TSG and ultrasonic‐assisted OSA–TSG were evaluated for toxicity at various levels using in vivo toxicological experiments following the methodology described by Puri et al. (2022). In the acute toxicity study, determination of LD_50_ was carried out in healthy rats. The present study was approved by the Institutional Ethics Committee of GCWUF, Pakistan. A complete experimental schedule is presented in Table 1, detailing a 21‐day study of acute oral toxicity. Initially, three rats were used in each treatment, and the starting dose of 300 mg/kg per body weight was administered orally using stainless steel oral gavage feeding needles. Two experimental groups were designated: Group_1_ (300 mg/kg TSG) and Group_2_ (300 mg/kg ultrasonic‐assisted OSA–TSG). The gums were dissolved in distilled water and administered as a single dose. No toxic effects, behavioral changes, or mortality were observed within 4 h of post‐administration. Following the initial assessment, the dose was escalated to 2000 mg/kg per body weight. Two additional groups were then treated: *G*_11_ (2000 mg/kg TSG) and *G*_22_ (2000 mg/kg ultrasonic‐assisted OSA TSG), in accordance with standard protocols. An additional group was designated as *G*_0_ (control). On the final day of the investigation, significant traits, including hematological and biochemical parameters, were determined; the cervixes of the rats were dislocated for the histological analysis of several organs before all living rats were put to death using mild anesthesia.

**TABLE 1:** Diet plan biosafety assessment.

Groups	Description
*G*_0_	Basal diet
*G*_1_	Pure TSG 300 mg/kg per body weight
*G*_2_	Modified TSG 300 mg/kg per body weight
*G*_11_	Pure TSG 2000 mg/kg per body weight
*G*_22_	Modified TSG 2000 mg/kg per body weight

#### 2.7.1 Physiological Test Parameters

Animals were monitored at least twice daily to assess viability and once daily for cage‐side clinical observations. Comprehensive clinical examinations were conducted prior to the first treatment and subsequently on a weekly basis until the conclusion of the study. Body weight and feed consumption were recorded during the acclimation period, on day 0 (first day of treatment), weekly thereafter, and immediately prior to necropsy.

#### 2.7.2 Hematological and Biochemical Test Parameters

On the final day of the treatment regimen, blood samples were collected from rats in each group via the retro‐orbital plexus and transferred into EDTA‐coated vials for hematology and biochemical analyses. The hemoglobin concentration (Hb), red blood cell count (RBC), white blood cell (WBC), lymphocytes, neutrophils, eosinophils, monocytes, basophils, platelet count (PLC), mean corpuscular hemoglobin (MCH), mean corpuscular hemoglobin concentration (MCHC), and mean corpuscular volume (MCV) were all determined using the Eurocount‐TS Hematology Analyzer. Meanwhile, biochemical parameters including blood glucose level (BGL), total cholesterol level (TCL), and triglyceride level (TG) were assessed. Blood urea nitrogen (BUN) and blood creatinine level (BCL) for renal function and serum alkaline phosphatase (SALP), serum aspartate aminotransferase (SAST), and serum alanine aminotransferase (SALT) enzyme level for liver function were determined (Heimbach et al. 2013).

#### 2.7.3 Histological Examination

On the 21st day of this experiment, all rats were euthanized under mild anesthesia, followed by cervical dislocation. Vital organs including the heart, brain, liver, and kidney were removed and immediately preserved in 10% v/v neutral buffered formalin. Alcohol dissolved in xylene was used to dehydrate all organs before they were embedded in paraffin for histological examination. Tissue sections were subsequently stained with hematoxylin and eosin to facilitate the visualization of pathological changes under a light microscope (Puri et al. 2022).

## 3 Result and Discussion

### 3.1 Extraction Yield

The tamarind seed gum yield was 36%. The instant result is consistent with the outcomes of Hettiarachchi et al. (2023), Gaur and Parvez (2019) and Chawananorasest et al. (2016), who recorded 35%, 38%, and 31% yield of tamarind seed gum, respectively. Similarly, the instant observation is comparable with the research of Crispín‐Isidro et al. (2019) and Mohamed et al. (2015); they indicated that variation in the yield was due to time and temperature of extraction techniques as well as varietal differences.

Ultrasonication is highly efficient in facilitating the substitution reaction during OSA modification of tamarind seed gum, as it enhances mass transfer through cavitation, disrupts intermolecular interactions, and exposes more hydroxyl groups for esterification. The localized high temperature and pressure generated by ultrasonic waves accelerate molecular mobility, lower activation energy, and significantly increase the reaction rate compared to conventional methods. This process often results in a higher degree of substitution (up to 1.5–2 times greater) with reduced chemical usage and shorter reaction times (by 30%–50%), while also improving key functional properties such as emulsification, oil‐holding capacity, and overall stability (Naeem et al. 2025).

### 3.2 Purity Analysis of TSG

The qualitative analysis confirmed the presence of carbohydrates in TSG while proteins, lipids, saponins, and tannins were not detected (Table 2). These components were effectively separated from the refined gum using various techniques. In this context, decortication removed the saponins and tannins from seeds. Moreover, lipids and proteins were separated through solvent extraction and the alkalization process. The investigation of Malviya et al. (2021) and Manimaran et al. (2022) expounded that TSG is predominantly composedof of carbohydrates. Comparable results were also observed in a study by Raj et al. (2015) which investigated the composition of *Azadirachta indica* gum.

**TABLE 2:** Purity analysis of TSG.

Tests	Outcomes	Confirmation
Benedict's test	Brick red precipitate	Carbohydrate Present
Sudan red III test	The color remained unchanged	Fat absent
Biuret test	Red color	Protein absent
Ferric chloride test	No greenish precipitate obtained	Tannins absent
Foam test	No foam formation occur	Saponins absent

### 3.3 Degree of Substitution

The degree of substitution measures the amount of hydroxyl groups relieved per monosaccharide unit and varies from 0 to 3 for polysaccharides (Zhang et al. 2024). The 0.0162 degree of substitution was observed in TSG_OSA‐US_. The hydroxyl groups of polysaccharides are static, so the reaction depended on how close OSA molecules are to them (Sweedman et al. 2013). The whole esterification process occurred largely in amorphous areas of the polysaccharide; that is why the crystalline structure remained unchanged (Shi et al. 2017). Gautam et al. (2023) revealed that ultrasonication significantly enhanced the chemical activity, quality, and degree of substitution of OSA‐modified starches.

### 3.4 Biosafety Assessment

During the experimental period, no morphological alterations or mortality were seen in the pure TSG and ultrasonic‐assisted OSA–TSG‐fed groups, which received oral single dosages of 300 and 2000 mg/kg. The animals fed both pure and modified gum were seen to be in good health. The group receiving high doses showed a slight declining trend in body weight gain when compared to controls (Table 3). However, the noted weight change was under the normal range between the control and gum fed groups. During the period of the investigation, no unusual alterations in behavior or ataxia, locomotor activity, or indicators of intoxication were noticed. Moreover, no alterations were noted in the fur coat, eyes, or respiratory system. Lastly, there were significant differences (*p* < 0.05) in the amount of food consumed by the treatment and control groups. A 21‐day period was chosen as a practical adaptation to evaluate early indicators of toxicity, metabolic tolerance, and physiological responses without extending to a full subchronic study. This duration is sufficient to capture potential initial adverse or cumulative effects, metabolic tolerance of OSA‐modified TSG consumption, indicating its reliability and safety for food application (Mahadevan et al. 2015).

**TABLE 3:** Physiological parameter for Sprague Dawley rats fed native and modified TSG for 21 days.

Physiological parameter	Day	*G*_0_	*G*_1_	*G*_11_	*G*_2_	*G*_22_
Weight (g)	0th	228.60 ± 1.01^a^	228.10 ± 1.27a	228.07 ± 0.90^a^	228.47 ± 1.25^a^	227.73 ± 1.04^a^
	21st	331.60 ± 1.10^a^	329.83 ± 1.30^ab^	326.33 ± 1.02^b^	331.07 ± 1.00^a^	328.27 ± 2.61^ab^
Feed intake (g/day)	0th	12.73 ± 0.10^a^	12.56 ± 0.11^a^	12.82 ± 0.18^a^	12.60 ± 0.13^a^	12.89 ± 0.24^a^
	21st	14.17 ± 0.10^a^	13.99 ± 0.09^ab^	13.86 ± 0.07^b^	14.04 ± 0.11^ab^	13.88 ± 0.06^b^

*Note:* Values within the same row with different superscript letters are significantly different (p < 0.05).

The findings of the hematological and biochemical parameters were all determined to fall within the normal range. ANOVA was used to evaluate the statistical data, and multiple comparisons were done by Tukey's test. The analysis test findings revealed that there was significant variation for Hb, RBC, WBC, lymphocytes, neutrophil, eosinophil, monocyte, basophil, and PLT in comparison to the control group. While a nonsignificant effect was found for MCH, MCHC, and MCV. The highest value for Hb, RBC, eosinophil, monocyte, PLT, MCHC, and MCV was found in the control group (*G*_0_), whereas the lowest value was found in the 2000 mg/kg ultrasonic‐assisted OSA TSG group (*G*_22_) for Hb, eosinophil, PLT, and MCHC, 300 mg/kg ultrasonic‐assisted OSA TSG group (*G*_2_) for RBC, and 2000 mg/kg TSG (*G*_11_) and *G*_22_ for monocyte and MCV. The highest value for WBC, lymphocyte, neutrophil, and basophil was found in *G*_22_, whereas the lowest was found in *G*_0_ for lymphocyte and neutrophil, and in *G*_0_ and 300 mg/kg TSG (*G*_1_) for WBC and basophil. The highest value for MCH was found in *G*_2_, whereas a lower value was found in *G*_22_ (Table 4). Data for serum biochemical parameters are presented in Table 5. There was a significant difference for BGL, TCL, TG, SAST, SALT, and BUN in groups where BCL showed a nonsignificant change within the groups. The highest value for BGL, TCL, TG, SAST, and BUN was found in *G*_0_, whereas the lowest value was found in *G*_11_ for BGL, TCL, and TG, *G*_22_ for SAST and BUN, and *G*_2_ for SALT. For SALP, the highest value was found in *G*_1_, and the lowest value was found in *G*_2_.

**TABLE 4:** Hematological parameters for Sprague Dawley rats fed native and modified TSG for 21 days.

Hematological parameters	Reference values	*G*_0_	*G*_1_	*G*_11_	*G*_2_	*G*_22_
Hb (g/dL)	10.4–16.5	14.20 ± 0.20^a^	13.90 ± 0.26^ab^	13.83 ± 0.10^ab^	14.00 ± 0.17^a^	13.51 ± 0.21^b^
RBC (×10^5^/μL)	38.0–66.8	64.50 ± 0.75^a^	63.46 ± 1.30^ab^	63.56 ± 0.65^ab^	63.43 ± 0.60^ab^	61.46 ± 0.65^b^
WBC (10^2^/μL)	44.00–148.00	72.10 ± 0.2^b^	72.10 ± 0.26^b^	72.33 ± 0.15^ab^	72.16 ± 0.25^ab^	72.70 ± 0.10^a^
Lymphocytes (%)	61–86	67.24 ± 0.07^b^	67.38 ± 0.09^ab^	67.47 ± 0.10^ab^	67.38 ± 0.08^ab^	67.49 ± 0.10^a^
Neutrophils (%)	13–36	13.91 ± 0.07^b^	13.97 ± 0.05^ab^	14.05 ± 0.06^ab^	14.06 ± 0.05^ab^	14.12 ± 0.06a
Eosinophils (%)	0–6	2.04 ± 0.03^a^	2.01 ± 0.02^ab^	1.99 ± 0.01^ab^	2.01 ± 0.01^ab^	1.98 ± 0.01^b^
Monocytes (%)	0–2	1.88 ± 0.02a	1.86 ± 0.01^ab^	1.84 ± 0.01b	1.86 ± 0.01ab	1.84 ± 0.01b
Basophils (%)	0–1	0.81 ± 0.01^b^	0.81 ± 0.01^b^	0.83 ± 0.01^ab^	0.82 ± 0.01^ab^	0.84 ± 0.01^a^
PLT (× 10^4^/μL)	17–55.7	35.84 ± 0.09^a^	35.82 ± 0.07^ab^	35.64 ± 0.08^ab^	35.8 ± 0.09^ab^	35.60 ± 0.08^b^
MCH (pg)	18.37–36.98	28.92 ± 0.06^a^	28.92 ± 0.06^a^	28.88 ± 0.03^a^	28.98 ± 0.02a	28.87 ± 0.02^a^
MCHC (g/dL)	25.41–80.55	32.36 ± 0.03^a^	32.35 ± 0.03^a^	32.36 ± 0.01^a^	32.35 ± 0.01^a^	32.40 ± 0.02^a^
MCV (fl)	29.41–12,307	53.86 ± 0.10^a^	53.74 ± 0.11^a^	53.64 ± 0.11^a^	53.73 ± 0.09^a^	53.64 ± 0.11^a^

*Note:* Values within the same row with different superscript letters are significantly different (p < 0.05).

**TABLE 5:** Biochemical parameters for Sprague Dawley rats fed native and modified TSG for 21 days.

Biochemical parameters	Reference values	*G*_0_	*G*_1_	*G*_11_	*G*_2_	*G*_22_
BGL (mg/dL)	62.4–201.8	122.20 ± 1.25^a^	113.73 ± 1.50^c^	105.23 ± 1.05^e^	117.83 ± 1.20^b^	109.67 ± 1.65^d^
TCL (mg/dL)	14.4–81.7	66.23 ± 1.15^a^	63.80 ± 1.15^ab^	62.33 ± 0.80^b^	66.06 ± 1.46^a^	65.46 ± 1.35^ab^
TG (mg/dL)	2.7–67.8	53.50 ± 5.58^a^	46.46 ± 5.85^ab^	31.70 ± 7.40^b^	50.86 ± 8.09^a^	45.50 ± 5.15^ab^
SALP (U/L)	160.8–838.3	213.93 ± 3.10^ab^	214.17 ± 2.79^ab^	220.10 ± 1.65^a^	213.13 ± 1.77^b^	210.77 ± 2.65^b^
SAST (U/L)	0.2–838.3	101.93 ± 1.65^a^	99.46 ± 1.20^ab^	98.31 ± 1.10^ab^	98.03 ± 1.50^b^	97.63 ± 1.65^b^
SALT (U/L)	1–223.3	45.93 ± 1.45^a^	43.46 ± 1.20^ab^	42.31 ± 1.12^ab^	42.10 ± 1.40^b^	41.53 ± 1.66^b^
BUN (mg/dL)	17.26–45.12	23.80 ± 1.01^a^	21.53 ± 0.85^ab^	21.43 ± 0.83^ab^	20.63 ± 1.15^b^	20.36 ± 1.15^b^
BCL (mg/dL)	0.2–1.2	1.15 ± 0.07^a^	1.30 ± 0.10^a^	1.10 ± 0.10^a^	1.10 ± 0.10^a^	1.10 ± 0.10^a^

*Note:* Values within the same row with different superscript letters are significantly different (p < 0.05).

**FIGURE 1:**
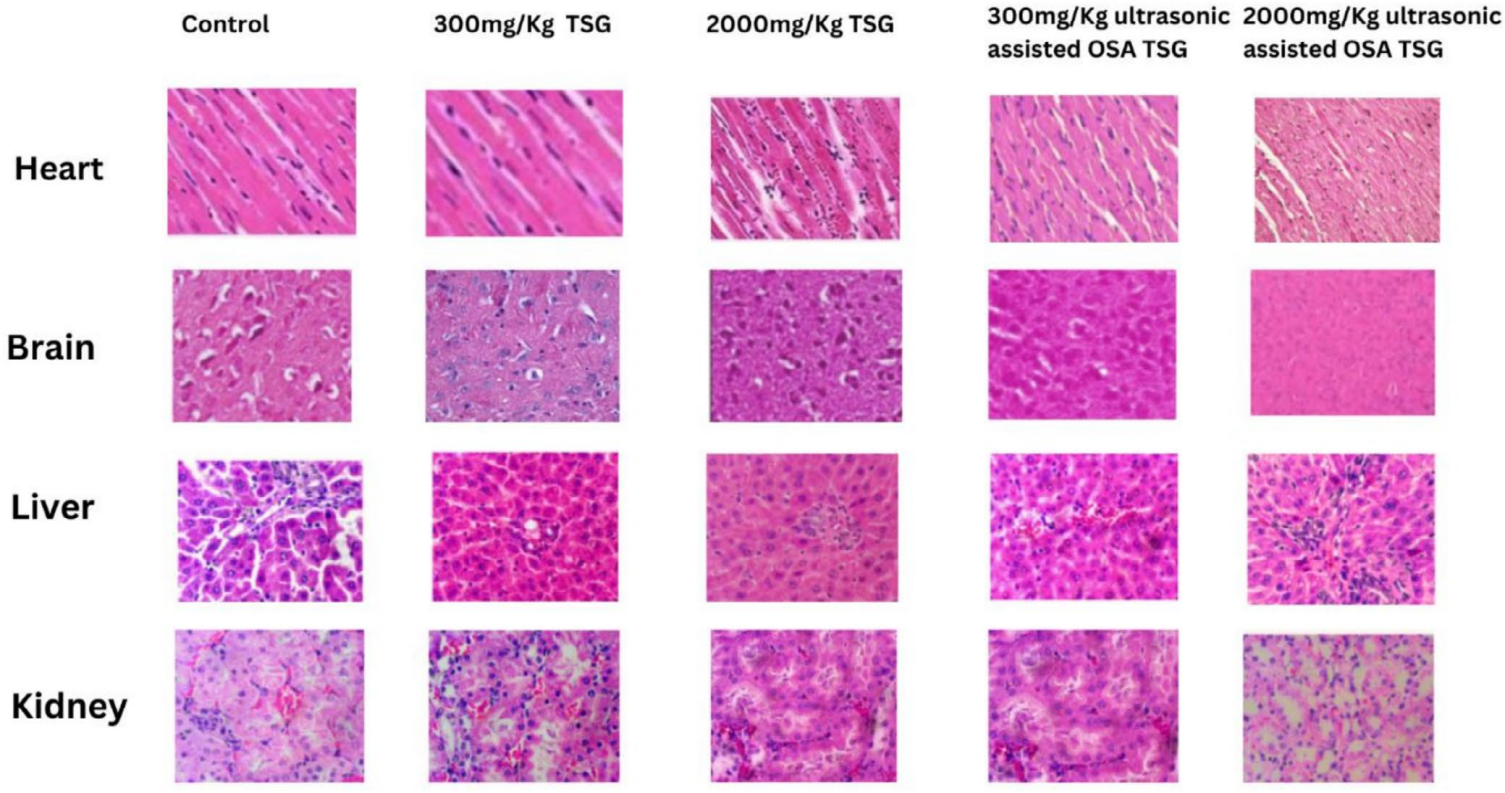
Histological analysis of Sprague Dawley rats fed native and modified TSG for 21 days.

According to the data, the heart, brain, liver, and kidney of the Spargue Dawley rats fed pure TSG and ultrasonic‐assisted OSA–TSG did not exhibit any notable histological alterations, as seen in Figure 1. It was found that the myocardial interstitial tissue and the heart's layers (the epicardium, endocardium, and myocardium) were normal. There was no change shown in the brain cells as compared to control. Furthermore, it was determined that the hepatic lobule, hepatic cell, and sinusoidal plate structures in the liver were all normal. Additionally, the Bowman's capsule encased the renal corpuscles, and the lining of the kidney's tubules, comprised of cuboidal epithelial cells, were comparable to control. The aforementioned results showed that there were no appreciable negative effects on the vital organs of Spargue Dawley rats when oral administration of single doses of TSG and ultrasonic‐aided OSA–TSG (300 and 2000 mg/kg) were used for acute toxicity studies.

When polysaccharides like starches and gums are modified using octenyl succinic anhydride (OSA), hydrophobic groups are added to improve their functional qualities without causing toxicity. A minimal fraction of hydroxyl groups in the polysaccharide is altered, maintaining the overall structure and function. Starch sodium octenyl succinate possesses a degree of substitution of less than 0.02, signifying negligible structural alterations (Sweedman et al. 2013). Similarly, gums' main chemical structure is not substantially changed by ultrasonic treatment. For example, studies on tara gum showed that ultrasound‐induced changes resulted in decreases in particle size and molecular weight without altering the basic chemical structures or functional groups. This implies that the gum's natural qualities are preserved, reducing the possibility of toxicity (Soares et al. 2023).

Extensive research has shown that dietary exposure to OSA‐modified polysaccharides does not lead to negative health consequences. According to Heimbach et al. (2013), the oral toxicity of tamarind seed polysaccharide was evaluated in Sprague Dawley rats at various dose levels; it was discovered that the rats' clinical chemistry and hematology did not change adversely as a result of the test substance's administration. The histological findings at necropsy showed no microscopic alterations. Likewise, OSA‐modified sago starch edible films were given to rats to check subacute oral toxicity. A nonsignificant effect was observed on body and internal organ weight, and feed intake indicated normal appetite. Biochemical test results showed that there were no negative impacts on kidney or liver function. Rats in the high‐dose group had elevated red blood cell counts, hematocrit, and hemoglobin, which may have been caused by decreased water intake rather than a toxic effect; however, other hematological parameters were all in the normal range. Histopathological analysis showed no appreciable tissue damage, confirming the safety of OSA‐modified starch at the investigated concentrations (Adzaly et al. 2023). Similar results were found by Mahadevan et al. (2014) and Puri et al. (2022) who determined the safety assessment of OSA‐modified starch on piglets and thiolated gum ghatti on rats.

## 4 Conclusions

The tamarind seed gum consisted entirely of carbohydrates and was further modified with the ultrasonic‐assisted OSA esterification method. For the safety assessment, both pure and modified gums were administrated to Sprague Dawley rats at a dietary level of 300 and 2000 mg/kg body weight for 3 weeks. No mortality was observed during the study. A slight but statistically significant decrease in body weight and feed intake was noted. The hematological and biochemical test results remained within the normal range. Moreover, histopathological analysis revealed no significant adverse effects on the vital organs of rats. Both types of gum were found to have LD50 greater than 2000 mg/kg body weight. Therefore, this study confirms that no adverse effects were observed following the dose administration of pure TSG and ultrasonic‐assisted OSA–TSG for 21 days.

## Author Contributions

**Adan Naeem**: wrote the original draft of the paper. **Rizwana Batool**: did the data curation and investigation. **Mahwash Aziz**: did the formal analysis and data curation. **Aysha Sameen**: did the investigation and formal analysis. **Aiza Zafar**: did the writing – review and editing. **Faiza Azmat**: did the validation and data curation. **Bakhtawar Saleem**: done the visualization and investigation. **Qamar Sajjad**: did the supervision and investigation. **Awais Raza**: did the data curation and the formal analysis. **Agoura Diantom**: done the writing – review and editing.

## Consent

All authors agree to publish.

## Conflicts of Interest

The authors declare no conflicts of interest.

## Data Availability

The data that support the findings of this study are available from the corresponding author upon reasonable request.
